# Metagenome-Assembled Genome of an Alphaproteobacterium Isolated from an HetDA Enrichment from the North Pacific Subtropical Gyre

**DOI:** 10.1128/mra.00595-22

**Published:** 2023-01-31

**Authors:** Martin Santillan, Elaina D. Graham, John F. Heidelberg, Eric A. Webb

**Affiliations:** a Department of Biological Sciences, University of Southern California, Los Angeles, California, USA; Montana State University

## Abstract

Here, we present HetDA_MAG_SS10, a metagenome-assembled genome (MAG) from an enrichment of a heterocystous diazotroph originally living in association with *Trichodesmium* spp. obtained near Station ALOHA in the North Pacific Ocean. HetDA_MAG_SS10, an alphaproteobacterium in the order *Micavibrionales*, is proposed to be photoheterotrophic via rhodopsin and has the potential for dimethylsulfoniopropionate (DMSP) demethylation.

## ANNOUNCEMENT

Here, we describe HetDA_MAG_SS10 a genome for a novel epibiont of the cyanobacterial HetDA enrichment. Samples were collected from the oligotrophic North Pacific Ocean near Station ALOHA, as detailed by Momper et al. (1). Briefly, *Trichodesmium* spp. were collected from the top 10 m with a 130-μm plankton net, and colonies were hand-picked and incubated in sterile YBC-II medium at 24°C with a 12-h light (100 μmol photons m^−2^ s^−1^)/12-h dark cycle. The enrichment was grown under the aforementioned conditions for 5 years prior to sequencing and was transferred to fresh medium every month. A 500-mL subsample of the enrichment was gravity filtered onto a 5.0-μm polycarbonate filter, and DNA was extracted using the Qiagen DNeasy PowerSoil kit. The DNA was sent to the University of Southern California Epigenome Center and sequenced on an Illumina MiSeq sequencer (paired-end 250-bp reads, with a total of 4,725,335 raw reads) using the MiSeq reagent kit v2 with 300 cycles. Reads were trimmed using Trimmomatic v0.38 (parameters: –phred33, ILLUMINACLIP:TruSeq3-PE.fa:2:30:10 SLIDINGWINDOW:10:28 MINLEN:50) (2). These reads were subsampled at the following percentages: 1, 3, 5, 10, 25, 30, and 50. Subsampled reads were assembled using MEGAHIT v1.2.8 (3). As described by Tully et al. (4), primary contigs from initial MEGAHIT runs were passed through CD-HIT-EST v4.8.1 (parameters: –M 500000 –c 0.99 –n 10) to identify overlapping primary contigs (5), followed by a secondary assembly in Minimus2 (AMOS v3.1.0) (parameters: –D OVERLAP = 1000 MINID = 98) (6). The Minimus2-generated contigs and the primary contigs that did not assemble with Minimus2 were combined to create the final contigs. The reads were mapped using Bowtie2 v2.3.5 (7) and filtered using CoverM v0.4.0 (–min-read-percent-identity 0.95 –min-read-aligned-percent 0.75) to calculate coverage (8). The assembly was binned using MetaBat2 v2.12.1 (9), BinSanity-wf v0.3.8 (10), and Concoct v1.1.0 (11) using default parameters for each, and DASTool v1.1.2 (12) was used to determine a final set of metagenome-assembled genomes (MAGs). All bins were run through MetaSanity v1.3.0 (13), FuncSanity, and PhyloSanity to provide annotations, using the default configurations and provided databases.

The closest relative of HetDA_MAG_SS10, with an average nucleotide identity (ANI) value of 77.19%, was *Micavibrionales* strain TMED2 (GenBank assembly accession number GCA_002168225.1), and HetDA_MAG_SS10 was determined to be novel via GTDB-tk (14)-generated ANI and relative evolutionary divergence (RED) scores. It has 16 contigs, a genome size of 3,186,557 bp, an *N*_50_ value of 457,653 bp, a coding density of 91%, and a GC content of 57.9%. It is 99.57% complete with 2.17% redundancy, as assessed using CheckM (15), and falls in the *Alphaproteobacteria* order *Micavibrionales.*

HetDA_MAG_SS10 contains most of the genes required for glycolysis and the tricarboxylic acid (TCA) cycle and lacks ribulose-1,5-bisphosphate carboxylase-oxygenase (RuBisCo). It contains the cytochrome *bd* complex (KEGG Orthology codes K00425 and K00426), which is associated with microaerobic respiration (16). Based on the presence of retinal biosynthesis genes (KEGG Orthology codes K06443, K02291, and K10027), β-carotene 15,15′-monooxygenase (TIGRFAM accession number TIGR03753), and rhodopsin (PFAM accession number PF01036), this organism is predicted to be capable of photoheterotrophy. HetDA_MAG_SS10 may also play a role in sulfur cycling, because it contains the *dmdA* gene for dimethylsulfoniopropionate (DMSP) demethylase, which is involved in DMSP degradation (17).

In summary, our MAG is predicted to be photoheterotrophic and to have roles in sulfur cycling, with its nearest relatives being obligatory parasites.

### Data availability.

Raw sequences and MAGs are available under BioProject accession number PRJNA719568. Raw reads are available in the SRA under accession number SRR14140256. The genome is available under BioSample accession number SAMN18613316.
